# COVID-19 Pandemic & Skin Care Guidelines for Health Care Professionals

**DOI:** 10.12669/pjms.36.COVID19-S4.2748

**Published:** 2020-05

**Authors:** Sadia Masood, Saadia Tabassum, Shaheen Naveed, Palwasha Jalil

**Affiliations:** 1 Dr. Sadia Masood, MBBS, FCPS, MHPE. Assistant professor & Section Head Dermatology, Department of Medicine, Aga Khan University Hospital, Karachi, Pakistan; 2 Dr. Saadia Tabassum, MBBS, FCPS Assistant Professor & Dermatology Program Director, Department of Medicine, Aga Khan University Hospital, Karachi, Pakistan; 3 Dr. Shaheen Naveed, MBBS, FCPS. Assistant Professor Dermatology, Department of Dermatology, Liaquat National Hospital, Karachi, Pakistan; 4 Palwasha Jalil, MBBS. Chief Resident Dermatology, Department of Medicine, Aga Khan University Hospital, Karachi, Pakistan

**Keywords:** Corona, Dermatitis, Skin care, Protective equipment

## Abstract

The Novel corona virus is bringing multiple challenges for health care professionals. Skin is the biggest organ and the first line of defense against different infections and external factors. Being the front line warriors, health care professionals are susceptible to various skin conditions due to prolonged use of personal protective equipment. These adverse skin conditions are redness, irritation, itching, contact dermatitis, and aggravation of underlying skin conditions like seborrheic dermatitis and acne vulgaris. In the current global situation, the potential incidence of such adverse dermatological effects does not in any manner cause the HCPs to deviate from the strict specific precautionary hygiene rules. These skin problems are manageable with the few precautionary measures. This article explores the different skin conditions that result from personal hygiene measures and usage of protective gear and will suggest some practical advice about how to manage and protect from these different adverse skin conditions.

The novel coronavirus is a new virus that has never been previously identified and it is spreading rapidly around the globe, outpacing the ability and resources of international health care systems. This outbreak was initially reported from Wuhan, China as a cluster of unknown respiratory illnesses.^1^ The disease reached Pakistan on February 26^th^ this year, when a patient was admitted with fever and respiratory symptoms.^2^ It is a highly contagious virus that spreads through the respiratory route, mainly by sneezing, coughing, through droplets from infected people, contact with contaminated surfaces, and community transmission. The spread of infection can be reduced through the use of proper protective equipment (PPE), the practice of maintaining diligent hand hygiene and social distancing.^3^

The new coronavirus creates a challenging environment for all physicians and health care workers, affecting their financial, personal, and social lives. Skin is the largest organ of our body and skin problems account for a substantial proportion of workplace injury and days away from work.^4^,^5^ Various skin conditions are emerging as a result of prolonged use of personal protective gear and extra personal hygiene measures. Skin manifestations in health care professionals (HCPs) are mainly due to skin friction, hyper-hydration effects, and contact reactions.^6^ These factors can also sometimes aggravate existing skin diseases. Commonly reported skin problems among HCPs due to PPE use include redness, scaling, itching, irritation, and skin maceration. Wearing a mask can cause pressure urticarial, contact dermatitis, itching, indentations, and acneiform eruption (Fig.1). The protective caps can cause scalp occlusion leading to itching, folliculitis and exacerbate seborrheic dermatitis. The nasal bridge is the most commonly affected skin site due to the continuous use of protective goggles. The use of latex gloves leads to occlusion, blister formation, sometime maceration that cause contact dermatitis (Fig.2). Excessive and frequent hand washing using detergents and disinfectants affects the hydro-lipid barrier may cause dryness and irritation.

**Fig.1:**
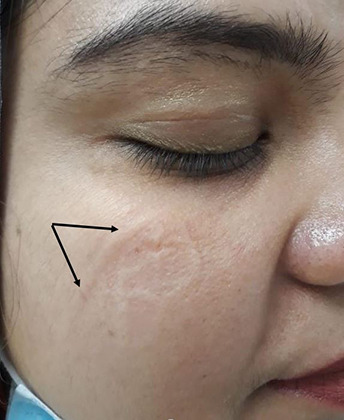
Skin indentations on cheek after using N-95 mask.

**Fig.2:**
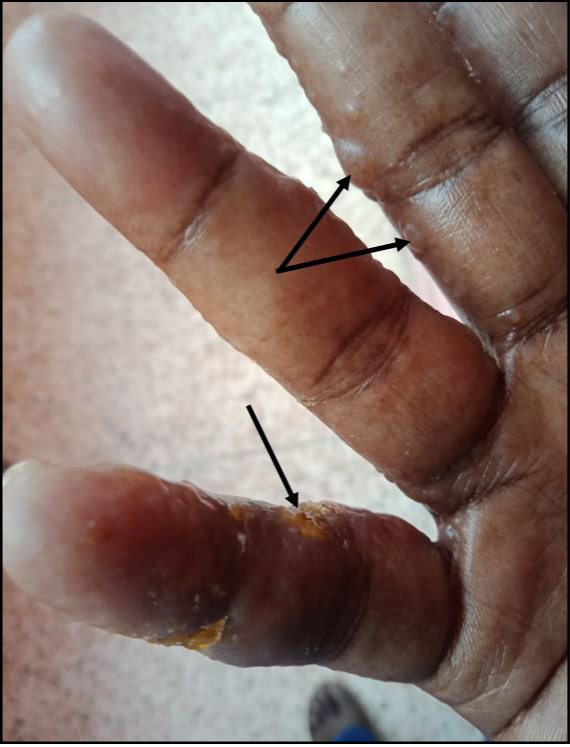
Clinical feature of irritant contact dermatitis on hands after prolong use of latex gloves (Shows multiple vesicles and erosions on fingers).

There is a very high prevalence of protective equipment related skin diseases. Despite the problem recognition, there is a paucity of data published on effective measures to decrease the incidence of these different skin conditions among health care professionals.^7^-^8^ The HCPs, working on the frontline against Corona Virus are more vulnerable to skin protective barrier damage. They should be able to recognize the consequences of wearing these protective gear and how to protect themselves from such issues.^1^,^9^ The following preventive measure is the best approach to avoid skin irritation, erosion, maceration, and contact dermatitis due to PPE and personal hygiene measure during the current pandemic:

- Skin scaling and dryness is very common because humid and close environments due to water in exhaled air can impair the skin barrier function. The application of good barrier cream (e.g Vaseline, petroleum jelly) before and after wearing PPE prevents such discomfort.^10^
- Wear a properly fitted mask and apply moisturizers on the face before wearing protective equipment. It would help to lubricate the skin and reduce skin friction. Wearing masks can cause skin indentations, which usually regress spontaneously.
- For persistent itching and pruritus at the site of the mask, put two to three layers of gauze inside the mask. and later wash face with tap water.
- Always moisturize the lips with good quality paraffin balm or petroleum jelly, before and after using PPE, as lip dryness can lead to cracking and proliferation of bacteria.
- For oral mucosal care, always try to rinse the mouth with normal saline or water when getting off from work.
- Try to use best-fitted goggles. Tightly fitted goggles cannot increase the protective effect, but instead, can generate fogs and damage the skin.
- Try to avoid washing your face with hot water or any irritant like ethanol, as it causes redness and swelling.
- For acne-prone skin, it is advisable to use moisturizers that contain oil control ingredients.
- A single layer of high quality latex gloves is sufficient to protect the skin. An extra layer is recommended if there is a risk of gloves breakage or any underlying existing skin condition.
- Apply good hand cream regularly and avoid wearing gloves for a longer time period. The use of cotton gloves inside latex gloves would help to protect against itching or irritation for those with latex allergy.^11^
- Frequent hand washing and the use of sanitizers can aggravate the existing hand dermatitis. Moisturizers and mild topical steroid creams can help the condition. It is important to change the hand-washing habits using mild soap to ensure a successful defense against virus spread and at the same time lowering the risk of adverse skin reaction.^11^
- Use sanitizers containing ethanol as the main component for hands hygiene.
- Keep your hair short, and try to cover hair completely with a surgical cap.
- Wash hair first before taking a body shower and avoid overheated water. If hairs are not contaminated then try to use normal, ordinary shampoo and avoid strong chemicals.
- Avoid excessive sweating as it tends to damage the skin barrier. It can be controlled by avoiding prolonged working hours, taking a proper shower after leaving the contaminated areas.
- Avoid showers with over hot water and apply moisturizers generally after every shower.
- Frequent showering further leads to surface lipids removal and a subsequent loss of stratum corneum, that may lead to scaling, itching, and dryness.
- Strictly avoid touching the periorbital regions and eyes with gloves or contaminated hands, follow the PPE guidelines of donning and doffing.
- Try to avoid the lips from contacting the contaminated site of masks while doffing and avoid touching lips until strict hand disinfection.
- If these skin conditions persist or worsened gradually, it is necessary to visit a dermatologist.

## CONCLUSION

Skin is the first line of defense and barrier against different types of infections and its integrity is lost due to various external factors. In the current global circumstances, the potential incidence of such adverse dermatological effects does not in any manner cause the HCPs and our general population deviating from the strict specific precautionary hygiene rules. These skin problems are manageable with the few mentioned precautionary measures. This article aims at exploring the possible detrimental dermatological conditions that can result from personal hygiene measures and usage of PPE as well as some practical advice for preventing these irritable skin conditions.

### Authors’ Contribution

**SM:** Concept, design, analysis and, revising it critically, is also responsible for final approval of the version to be published

**ST:** Contributed significantly in concept of study, and revised it critically, final approval of the manuscript.

**SN:** Contributed in the concept, analysis and critical review of article.

**PJ:** Contributed in critical analysis and preparation of the manuscript.
